# Method comparison for Japanese encephalitis virus detection in samples collected from the Indo-Pacific region

**DOI:** 10.3389/fpubh.2022.1051754

**Published:** 2022-11-24

**Authors:** Gary Crispell, Kelly Williams, Eric Zielinski, Akira Iwami, Zachary Homas, Karen Thomas

**Affiliations:** Environmental Molecular Biology Laboratory (EMBL), Division of Laboratory Sciences, US Army Public Health Command – Pacific, Zama-shi, Japan

**Keywords:** Japanese encephalitis virus (JEV), Magpix, SISPA, minION device, MiSeq, Twist Comprehensive Viral Research Panel (CVRP)

## Abstract

**Introduction:**

Japanese encephalitis virus (JEV) is a mosquito-borne viral pathogen, which is becoming a growing public health concern throughout the Indo-Pacific. Five genotypes of JEV have been identified. Current vaccines are based on genotype III and provide a high degree of protection for four of the five known genotypes.

**Methods:**

RT-PCR, Magpix, Twist Biosciences Comprehensive Viral Research Panel (CVRP), and SISPA methods were used to detect JEV from mosquito samples collected in South Korea during 2021. These methods were compared to determine which method would be most effective for biosurveillance in the Indo-Pacific region.

**Results:**

Our data showed that RT-PCR, Twist CVRP, and SISPA methods were all able to detect JEV genotype I, however, the proprietary Magpix panel was only able to detect JEV genotype III. Use of minION sequencing for pathogen detection in arthropod samples will require further method development.

**Conclusion:**

Biosurveillance of vectorborne pathogens remains an area of concern throughout the Indo-Pacific. RT-PCR was the most cost effective method used in the study, but TWIST CVRP allows for the identification of over 3,100 viral genomes. Further research and comparisons will be conducted to ensure optimal methods are used for large scale biosurveillance.

## Introduction

Japanese encephalitis virus (JEV) is a mosquito-borne pathogen, which is the causative agent of Japanese encephalitis, and has become a growing public health concern throughout Asia. JEV is a flavivirus with five identified genotypes (I, II, III, VI, and V) (1). JEV genotype I is currently the most prevalent genotype present throughout Asia and the Indo-Pacific. JEV genotype III was the prevailing genotype until genotype I displaced it in the 1990's (2). JEV is primarily vectored by *Culex (Cx.) tritaeniorhynchus*, but has been shown to be present and potentially transmitted through various other *Culex* species and *Aedes (Ae.) japonicus* (3). In fact, a thorough review of current research on JEV vector competency determined 14 species of mosquitoes to be confirmed vectors of JEV: *Ae. albopictus, Ae. vexans, Ae. vigilax, Armigeres (Ar.) subalbatus, Cx. annulirostris, Cx. bitaeniorhynchus, Cx. fuscocephala, Cx. gelidus, Cx. pipiens, Cx. pseudovishnui, Cx. quinquefasciatus, Cx. sitiens, Cx. tritaeniorhyncus* and *Cx. vishnui* (4). Mosquito vectors acquire viruses from animal reservoirs, mainly pigs and birds (5). In this fashion, the viruses found in pigs are commonly farmed, and can present risks to nearby human populations by providing mosquitos an amplifying host for acquiring JEV, and pigs show high viremia for 4–5 days after initial infection (6). However, there are also reports of JEV cases on Japanese islands farther away from the main island, where there are no pig farms. It is possible that in these circumstances that wild boar are the reservoir/amplifying host (7). In South Korea, early May to late October is the peak activity season for the *Cx. tritaeniorhynchus* species and it is when there is the greatest risk of contracting this disease (8). In a recent study, 274 *Cx. tritaeniorhynchus* mosquitos successfully fed on an animal that was infected with JEV, molecular analysis showed that 261 out of the 274 mosquitoes were found to contain JEV (9).

Most reported cases of JEV infections are asymptomatic or have non-specific febrile symptoms after 5–15 days incubation period (10). JEV occasionally leads to severe disease, manifesting in non-cell necrotic plaques with edema, bleeding, inflammatory infiltration in multiple regions of the brain, and severe neurologic sequelae increasing the childhood morbidity and mortality (11). Case numbers of Japanese encephalitis (JE) vary from country to country, with the World Health Organization estimating that there are ~68,000 cases of JE each year world-wide (12). JE primarily occurs in children, with adults in endemic countries showing immunity from childhood infection. However, it is possible to contract the disease at any age. The morbidity rate is <1% of the infected population, but once a patient has on-set of symptoms, there is no specific treatment, and the main treatment is supportive therapy (7, 10). JEV prevention measures include the use of personal protective gear like insect repellents, nets, and long sleeved clothing.

Vaccination is the most effective means of preventing JEV infection (13). Past studies have shown that vaccination against Japanese encephalitis is an important and effective tool for prevention of disease. The cross protective capacity against other genotypes has been studied for the current Japanese encephalitis vaccine, a vaccine based on genotype III which may prevent infection for other genotypes I–IV (14). Sequence comparisons of antigenic regions of genotypes I–V indicate nucleotide (nt) similarities of <90% for JEV genotype I and <80% for JEV genotype V as compared to JEV genotype III (15). Serum studies have shown, JEV genotype III inactivated vaccines neutralize JEV genotype I viruses, but with reduced efficiency (16).

A 2015 study performed in the Republic of Korea tested the serum of 1,000 soldiers that had never previously deployed to determine the prevalence of JEV antibodies among personnel in the U.S. Forces Korea (17). They found that prevalence was as low as 0.2% of the population studied, and at least one soldier had titers high enough to indicate current or recent infection.

A 2018 mosquito surveillance project conducted in Camp Humphreys, ROK sequenced 6,540 *Culex* spp. in 260 pools. Analysis revealed 122 distinct virus species in the pools (15). Two of these pools were positive for JEV genotype V along with other viruses. Discovery of JEV genotype V in a highly populated area adjacent to Seoul is of concern, due to questions regarding vaccine effectiveness against JEV genotype V. Currently, it is recommended by the U.S. Centers for Disease Control and Prevention that travelers to JE-endemic countries receive a 2-dose series of IXIARO prior to travel, particularly for those travelers with longer plans (greater than a 1 month stay), those who will frequently travel to JEV-endemic countries, or those with shorter stays that may be at greater risk due to planned activities, season of travel, or type of accommodation during stay (18). Given the potential serious outcomes and effect on force readiness, it is of great concern for the public health of both military and civilian populations to accurately detect JEV to ensure the safety of our military populations.

Viral encephalopathies continue to be a public health concern in many parts of the world. In this study, we examine and compare various detection methods for Japanese encephalitis virus to determine which are the most sensitive, cost effective, and provide the most data to the investigating scientists. The methods covered are RT-PCR, Luminex MagPix technology, the TWIST Comprehensive Viral Research Panel, direct MinION sequencing, and a SISPA method utilized with both the MinION and MiSeq.

## Materials and methods

### Mosquito identification and grouping

Mosquito samples were captured using a Mosquito Magnet (Woodstream Corporation, Lancaster, PA, USA) trapping device and then sorted and identified by the submitting entomologist according to collection date, collection site (including location coordinates), and species. Specimen pools consisted of varying numbers of mosquitoes (1–30) in each microcentrifuge tube. Collections that exceeded more than 30 specimens were sorted in to multiple tubes. Mosquito pools were maintained at −80°C until the time of homogenization and RNA isolation.

### Materials

All common laboratory supplies and reagents were obtained through Thermo Fisher Scientific (Waltham, MA, USA) and its subsidiaries unless specifically noted. Molecular grade isopropanol and ethanol were sourced locally through Kanto Chemical Co. (Chuo-ku, Tokyo, Japan).

### RNA isolation

Frozen mosquito samples stored in microcentrifuge tubes were allowed to thaw at room temperature for up to 15 min. Using the Zymo Direct-zol™-96 MagBead RNA kit (Zymo Research, Irvine, CA, USA), 400 μL of TRI reagent and two RNase-free 3.2 mm stainless steel beads (Next Advance, Troy, NY, USA) were added to each specimen tube. Samples were lysed using the Qiagen TissueLyser II for 7 min at a frequency of 24/s followed by centrifugation for 10 min at 14,000 rcf. Two hundred microliters of supernatant was removed from each sample tube and pipetted into a 96-deep well plate. Two hundred microliters of 99.5% ethanol, 20 μL of MagBinding beads, and 5 μL of Proteinase K were added to each sample well before adding the plate to the Thermo Scientific KingFisher Flex automated instrument. An extraction control, consisting of all reagents and no mosquito matrix, was added to the sample plate as well. The elution plate was stored at −20°C for short-term storage or used immediately for further analysis on the Applied Biosystems ABI 7500 Fast DX, MagPix, MinION and MiSeq. A second extraction was performed on the homogenized mosquitos with the addition of 50 μL DNase added to the sample prior to being placed on the Thermo Scientific KingFisher Flex automated instrument.

### RT-PCR

RT-PCR was performed within the specifications provided by the Applied Biosystems TaqMan Fast Virus Master Mix protocols. Frozen reagents and RNAs were thawed on ice. The mastermix formula used was 5 μL of TaqMan Fast Virus, 1 μL of the 10 μM gene-specific forward and reverse primers, 0.80 μL of gene-specific probe at 5 μM (Table 1) and 7.20 μL of nuclease free water per reaction. Each reaction consisted of 15 μL of mastermix along with 5 μL of RNA. An extraction control, non-template control, and positive control were used for each plate. The following thermocycling conditions were used on the ABI 7500 Fast Dx instrument: 50°C for 5 min; 95°C for 20 s; 40 cycles of 95°C for 3 s and of 60°C for 30 s. The amplification plot and Ct values were used to determine the presence of Japanese Encephalitis Virus (JEV).

**Table 1:** Primers and probes used to detect and genotype JEV in this study.

**Japanese encephalitis primers and probes**		
**Name**	**Sequence**	**References**
JEV F	5′-GGCTCTTATCACGTTCTTCAAGTTT-3′	(19)
JEV R	5′-ACTAGTAAGTTTCATTGCCACACTCT-3′	(19)
JEV Probe	5′-ATTAGCCCCGACCAAGGCGCTTT-3′	(19)
JEV-G1/G3-F	5′-GGTCTGCAACCCAAACAAGAA-3′	(20)
JEV-G1/G3-R	5′-GCCAGCATGAAGGGTATTGACAT-3′	(20)
JEV-G1-Probe	5′-TTGTGGGAGGTCTAGCCGAGTTGG-3′	(20)
JEV-G3-Probe	5′-TCGTAGGTGGTTTGGCCGAGTTG-3′	(20)
JEV-G5-F	5′-TGCGACAAACAAGCCGTGTA-3′	(20)
JEV-G5-R	5′-TTGCACTGACACAGATCTTCTACTTCT-3′	(20)
JEV-G5-Probe	5′-CGTTGCACGAGGACCAGGCACTC-3′	(20)

### MagPix mega mosquito panel

RNA from positive samples, as detected by RT-PCR, were reacted in accordance to the instructions for use included with the GenArraytion Inc. MultiFLEXMega Mosquito Borne Panel (South Orange, NJ, USA). In the first step, RNA was converted into cDNA before PCR utilizing the Qiagen One-Step PCR Kit and panel-specific primers according to the instructions included with the panel. Additionally, five different controls from GenArraytion were run with the samples to ensure that all targets amplified as expected. Next, 10 μL of the PCR products was mixed with 5 μL of panel-specific beads and 35 μL Buffer A and beads were hybridized to the target DNA/cDNA according to instructions. Finally, the beads were placed on a magnetic plate and liquid removed *via* pipette. The beads were quickly taken off the magnetic plate and a streptavidin solution of 5 μL SAPE and 70 μL Buffer B was mixed with the beads and heated at 52°C to allow the beads to fluoresce in the MagPix instrument. Plates were maintained at 52°C and quickly entered onto the pre-heated instrument so that analysis could be conducted.

### Twist CVRP

Library preparation and target enrichment standard hybridization workflow followed the guidelines of Twist Next Generation Sequencing (NGS) protocols Twist Total Nucleic Acids Library Preparation EF Kit 2.0 for Viral Pathogen Detection and Characterization along with their Twist Target Enrichment Protocol. Three previously extracted RNA samples were diluted along with synthetic controls. cDNA synthesis and purification followed. Next, DNA fragmentation, telomere repair, and dA-Tailing were performed. Universal Twist adapters were then ligated to the cDNA and purified. Finally, PCR amplification was conducted to index the samples and finish the library preparation portion of the study. A single pooled library was first prepared from the indexed library-prepped sample pools. This was followed by hybridization of the targets in solution, which was ~16 h in total to complete. Next, the binding of hybridized targets to desired streptavidin beads occurred. Libraries were then enriched via PCR amplification and purification utilizing 23 cycles as recommended by Twist Technical Support. Sample libraries were ready for sequencing on the Illumina NGS platform according to manufacturer protocols after PCR amplification and purification. Sequencing data was processed on the One Codex bioinformatics online platform and according to methods described below.

### Sequence-independent, single-primer-amplification mediated MiSeq sequencing

Sequence-Independent, Single-Primer-Amplification (SISPA) is a method of tagged random amplification of nucleic acid targets that has been shown to be suitable for preparing samples for whole genome viral sequencing (21). Random hexamer tagging with 20 nt barcode “K” (GACCATCTAGCGACCTCCAC) was performed using primer K8N (GACCATCTAGCGACCTCCACNNNNNNNN) as described by Chrzastek et al. (22). Library preparation was performed with an optimized protocol provided by USAMRIID Center for Genome Sciences using the K/K8N primers as described above. In short, first strand synthesis is performed with primer K8N, dNTPs, RNA template and nuclease-free water. This is heated at 65°C for 5 min, placed on ice and then 5X SuperScript IV buffer, DTT, RNaseOUT Recombinant RNase Inhibitor are added to each library before incubating for 10 min each at 23, 50, and 80°C and held at 4°C. Second strand synthesis was performed with RNAseH and Klenow 3′ -> 5′ Exo DNA polymerase added to the first strand mix and incubated for 30 min at 37°C, 20 min at 75°C and held at 4°C. Cleanup was performed with AMPureXP beads according to protocol. Random fragment amplification was performed with primer K, 5x Phusion HF buffer, dNTPs, nuclease-free water, and Phusion Hot Start II DNA polymerase and template cDNA. This mix was cycled for 30 s at 98°C, 40 cycles of 10 s at 98°C, 10 s at 50°C, and 45 s at 72°C, and a final cycle of 72°C for 10 min before being held at 12°C. A new mix was made and added to each sample for a final cycle of 30 s at 98°C, 10 s at 50°C, and 72°C for 10 min and held at 12°C. PCR products were then cleaned using the AgenCourt Ampure XP Beads before moving to finishing library prep and loading on the MiSeq. PCR products were fragmented, tagged with adaptors, and appended with unique dual index sequences *via* secondary PCR using reagents from the NEBNext Ultra II Directional RNA Library Prep Kit for Illumina and NEBNext Multiplex Oligos for Illumina (Dual Index Primers Set 1) according to manufacturer protocol.

### MinION sequencing

Two methods of minION sequencing were attempted. Unprocessed whole RNA extract as well as SISPA mediated processing of RNA as described above were used to generate cDNA. The cDNA was then processed using the Nanopore Rapid Sequencing Kit SQK-RAD004 for library preparation. 7.5 μl of undiluted cDNA was used in the library preparation process to obtain as near to 400 ng of cDNA as possible. The rest of library prep and loading onto the minION was done according to manufacturer procedure.

### Bioinformatics

Sequence data was treated based on single or paired reads and aligned to target genomes and visually mapped with Tablet software (The James Hutton Institute, Scotland, UK). MinION FASTQ single reads were combined for single file processing using the concatenate (cat) command and FASTA files were generated using seqtk (23). MiSeq paired read data were first joined using FLASH (Fast length adjustment of short reads) for single file processing (24). FASTQ files were then fed into the NanoPipe and assembled and aligned against target genomes (25). Tablet software was used to view the resulting BAM files mapped against the target genome for visual coverage of the genome and mismatch data, see Supplementary Figure 1 (26). All sequencing data was uploaded to NCBI with BioProject accession PRJNA894324.

## Results

Of 549 mosquito pools, 21 pools were found to be positive, corresponding to a 3.8% positivity rate from *Cx. tritaeniorhynchus* as detected using described methods. A majority of the positive samples analyzed using the ABI 7500 Fast Dx platform and Fast Virus RT-PCR mastermix provided us with relatively low Ct values upon extraction (Table 2). Analyzing the positive samples with Qubit provided total RNA concentrations per sample. The Broad Range Qubit Assay was suitable for running samples with Ct values up to 20.0, and High Sensitivity Assay should be considered for samples with higher Ct values.

**Table 2:** JEV positive samples, “# in Pool” reflects how many mosquito specimens were in each sample pool tested, with their respective Ct values, and genotype.

**Sample**	**Species**	**# in Pool**	**Ct**	**JEV**	**JEV**	**JEV**
				**G1**	**G3**	**G5**
A21.2333	*Cx. tritaeniorhynchus*	21	16.79	✓	X	X
A21.2437	*Cx. tritaeniorhynchus*	30	18.61	✓	X	X
A21.2560	*Cx. tritaeniorhynchus*	30	18.61	✓	X	X
A21.2711	*Cx. tritaeniorhynchus*	30	34.99	✓	X	X
A21.2838	*Cx. tritaeniorhynchus*	30	20.29	✓	X	X
A21.2886	*Cx. tritaeniorhynchus*	30	18.67	✓	X	X
A21.3160	*Cx. tritaeniorhynchus*	30	24.52	✓	X	X
A21.3171	*Cx. tritaeniorhynchus*	30	20.53	✓	X	X
A21.3232	*Cx. tritaeniorhynchus*	19	18.82	✓	X	X
A21.3389	*Cx. tritaeniorhynchus*	30	33.84	✓	X	X
A21.3401	*Cx. tritaeniorhynchus*	30	24.29	✓	X	X
A21.3402	*Cx. tritaeniorhynchus*	30	19.48	✓	X	X
A21.3404	*Cx. tritaeniorhynchus*	30	30.55	✓	X	X
A21.3465	*Cx. tritaeniorhynchus*	30	19.10	✓	X	X
A21.3577	*Cx. tritaeniorhynchus*	30	20.08	✓	X	X
A21.3585	*Cx. tritaeniorhynchus*	30	29.63	✓	X	X
A21.3587	*Cx. tritaeniorhynchus*	30	18.12	✓	X	X
A21.3675	*Cx. tritaeniorhynchus*	30	17.70	✓	X	X
A21.3678	*Cx. tritaeniorhynchus*	30	23.29	✓	X	X
A21.3682	*Cx. tritaeniorhynchus*	30	23.31	✓	X	X
A21.3683	*Cx. tritaeniorhynchus*	30	18.62	✓	X	X
JEV 1:1,000	*Unknown*	N/A	26.65	X	✓	X

G1, genotype I; G3, genotype III; G5, genotype V.

Utilizing the primers and probes as shown in Table 1, the samples were then genotyped *via* RT-PCR. Table 2 shows that all 21 JEV positive samples produced sigmoidal curves when tested for JEV genotype I and none of the samples produced any curve when tested for JEV genotype III or V. The positive control used did not produce a sigmoidal curve for JEV genotype I, but did so for genotype III.

**Figure 1:**
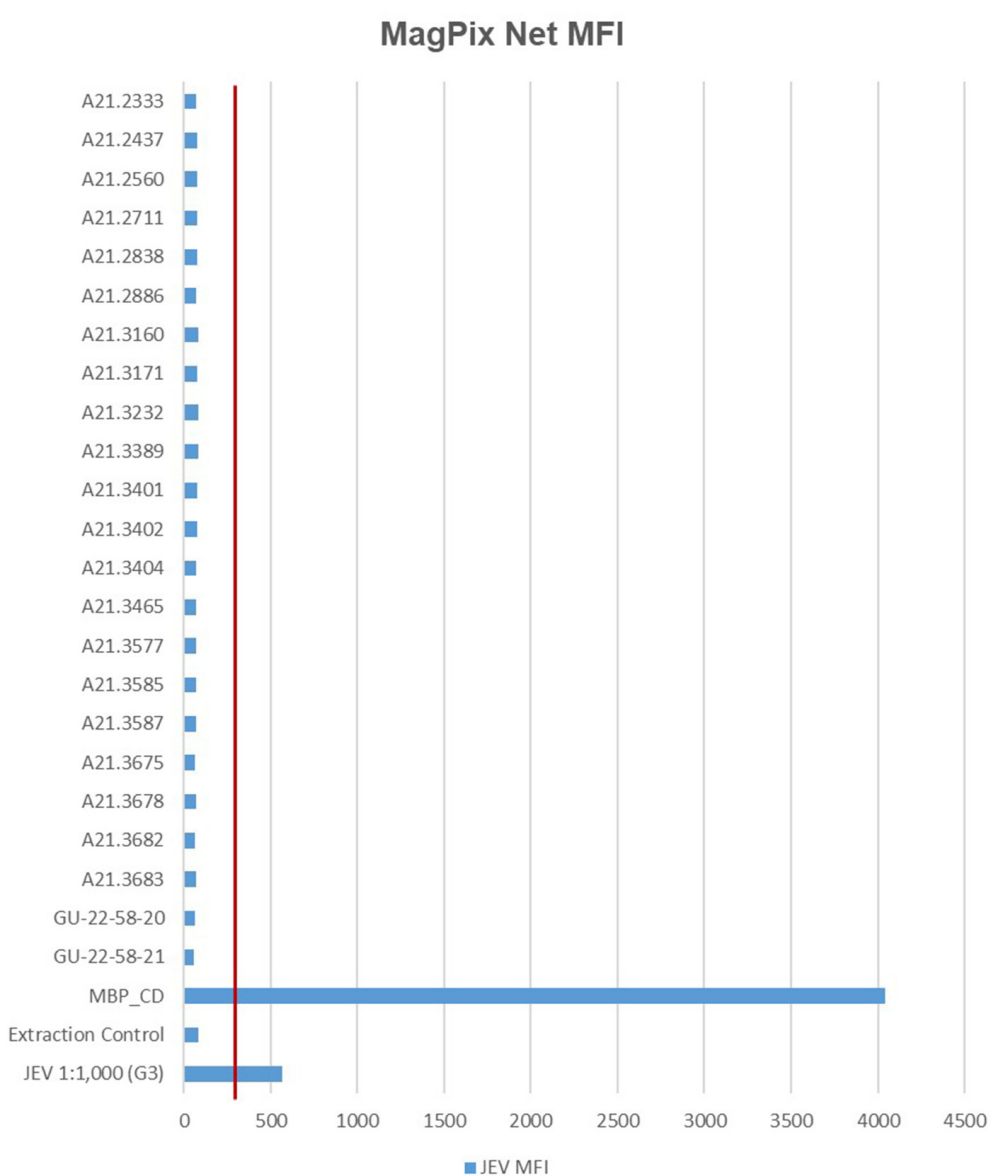
MFI values for MagPix MultiFLEX^®^ panel analytes. Red line represents 300 MFI; samples above 300 MFI are positive. Samples labeled A21.xxxx are positive for JEV as determined by RT-PCR. Samples labeled GU-22-58-xx are negative for JEV. JEV 1:1,000 (G3) is an archived sample. Internal Control, Fluorescence Control and Non-specific Binding Control had comparable values between specimen types. MFI, median fluorescent intensity; MBP_CD, positive control.

In total, 21 JEV positive sample pools and 21 JEV negative sample pools, were analyzed with the MagPix instrument using the GenArraytion Mega Mosquito-borne MultiFLEX^®^ Panel to assess for the presence of JEV, in addition a historical sample used as a RT-PCR control at a dilution of 1:1,000 was analyzed. The data in Figure 1 and Supplementary Table 1 show that the Net MFI was below 200, a negative result, for all JEV samples tested, with the positive control MBP_CD over 4,000 and the historic RT-PCR control for JEV at a 1:1,000 dilution was positive at over 500. A signal above 300 is considered a positive result for each analyte and below 200 is negative, per manufacturer's insert. All Internal, Fluorescence and Non-Specific Binding Controls were acceptable for samples and controls ran.

**Figure 2:**
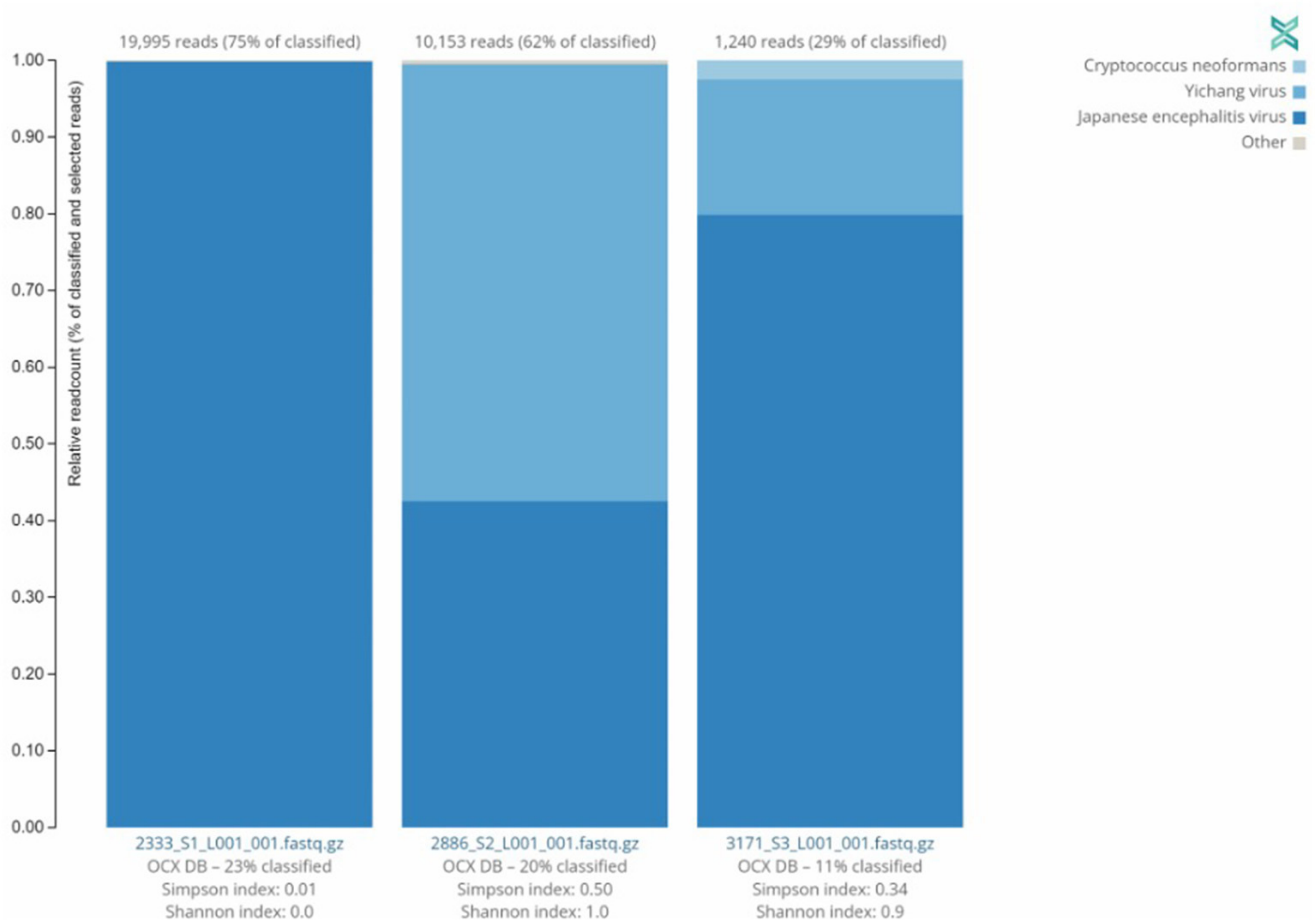
Distribution of sequences identified by Twist CVRP. Three of the JEV positive samples analyzed with the Twist Biosciences CVRP are shown with their read counts and individual distributions of Japanese encephalitis virus, Yichang virus, *Cryptococcus neoformans*, and other sequences.

Three (3) JEV positive samples were sequenced using the Twist Comprehensive Viral Research Panel (CVRP) on the Illumina MiSeq. All three samples were confirmed to contain JEV *via* sequencing, and additionally a mosquito specific virus, the Yichang virus, was detected in two of the three samples. One sample, sample 3,171, showed evidence of fungal contamination with the One Codex platform detecting the presence of Cryptococcus neoformans. MiSeq FASTQ files were also assembled and aligned to JEV Genotypes I (GenBank Accession JN381833.1), III (GenBank Accession KP164498.2), and V (GenBank Accessions HM596272.1 and JF915894.1). Comparisons indicated significant alignment to Genotype I vs. III and V with a Kruskal–Wallis test of mismatch percentage of each type showed a *p*-value of 0.009343. This information and the relative abundance are summarized in Table 3 and Figure 2 below.

**Table 3:** Bioinformatics results of TWIST CVRP and SISPA sequencing data.

**Sample**	**JEV G1**	**JEV G3**	**JEV G5 Muar**	**JEV G5 XZ0934**
**Twist mismatch percentages**				
A21.2333	2.7	9.3	17.1	17.4
A21.2886	3.1	10.7	18.9	19.0
A21.3171	3.4	10.2	18.0	18.2
**SISPA mismatch percentage**				
MinION pool 1	14.4	19.9	21.7	23.1
MinION pool 2	12.6	19.4	22.4	22.6
MiSeq pool 1	2.7*	9.3*	18.3*	17.7*

Sample names and mismatch percentage for each genotype. ^*^MiSeq Pool 1 results are estimates due to low number of aligned reads.

Direct minION sequencing of cDNA provided ample coverage of the Cx. tritaeniorhynchus mitochondrial genome, however, it was not able to provide any reads necessary to determine that JEV was present in the sample. SISPA mediated sequencing on the minION was able to provide near full genome coverage using NanoPipe. Analysis of the various genotypes (G1, G3, G5) showed agreement with Twist results, but with a higher mismatch percentage (Table 3). The resulting reads from the SISPA sequencing on the MiSeq were combined and processed. Very few reads were aligned to the JEV genomes, so accuracy of the mismatches cannot be assessed, however they appear to match Twist results (Table 3).

## Discussion

RT-PCR was the quickest, easiest, and most cost-effective method for detection of JEV in our study, requiring <2 h from setup to results. The ABI 7500 provided accurate and reproducible results for both genotypes I and III, while the MagPix Mega Mosquito panel was able to detect JEV genotype III from an archived sample, but not JEV genotype I in our recently extracted mosquito samples. Due to lack of Genotype II, IV, and V sample material, it has yet to be determined if the panel can detect the remaining JEV genotypes. For proper public health surveillance, our lab requires the ability to test for and detect all genotypes of JEV. Currently, the Mega Mosquito panel does not instill confidence to support the Public Health Command—Pacific's goal of vector-borne disease detection in high risk areas where JEV infection is possible, especially when considering lower throughput, increased analytical time compared to RT-PCR, and 10-times the analysis cost per pool. Because of these factors combined, future surveillance will be conducted using the RT-PCR centric approach and genotype identifications will be carried out using NGS based solutions. Future considerations for surveillance could also include analysis of reservoir hosts, such as wild boars and pigs, within a radius of 7.5 km of locations discovered to have JEV positive mosquitoes, which is the reported maximum flight range of *Cx. tritaeniorhynchus* (27).

This study focused primarily on the detection of JEV types I, III, and V as the majority of our arthropod samples originated in Eastern Asian countries and the Pacific Islands. There is conflicting data regarding if the current Japanese encephalitis vaccine, derived from G3 JEV, can induce protective immunity against the remaining genotypes. Furthermore, vaccine potency against the emerging G5 genotype has not yet been reported (28). More detailed studies demonstrating increased levels of protective antibodies against JEV Genotype V are needed. Research suggests that JEV genotype V re-emerged after nearly a half century hiatus. The first strain or the Muar strain was isolated in Malaya in 1952. The second was extracted from mosquito samples collected in China in 2009 and designated strain XZ0934. The emergence of JEV genotype V has been detected in the Republic of Korea (ROK) as recently as 2016–2018. A JEV genotype shift may be occurring in the ROK. Initially, the prevalent JEV genotype was identified as Genotype III until ~1990s when it shifted to Genotype I, and it may be shifting once again to Genotype V, which was first identified in 2010 (15). Therefore, delivering optimal surveillance detection methods against all predominant JEV genotypes in the surrounding area is critical for the overall wellbeing of both civilian and military personnel.

Identifying predominant genotypes by area will allow public health officials to make informed and more effective decisions concerning prevention, testing, and vaccine allocation/development. Utilizing the real-time PCR with universal and genotype specific primers/probes (Table 1), we successfully genotyped all positive samples as JEV genotype I as shown in Table 2. This supports current data indicating JEV genotype I is the predominant genotype within our testing areas of the Indo-Pacific. We further confirmed findings using the Twist Comprehensive Viral Research Panel on the Illumina MiSeq platform. OneCodex software provided for use with the Twist panel detected JEV, but did not provide typing information. However, we were able to use the sequencing data generated from the protocol to analyze *via* our own pipeline. Using a Kruskal–Wallis Rank Sum Test comparing the amount of aligned base pairs from each sample to each genotype, provided alignment data that suggested JEV genotype I. With a *p*-value of 0.009343, the differences in alignment between Type I and Types III and V were highly significant. This method is certainly useful for pathogen discovery, however, given the multiple days and hours of hands on bench time required as well as the very significant cost of reagents required for the protocol, we cannot recommend this method for routine screening for pathogens. Additionally, more optimization of the assay is required for higher sensitivity when analyzing arthropod samples, and possibly the use of an arthropod specific blocking agent. The number of cycles for the panel would also need to be adjusted from manufacturer recommendations to ensure maximum optimization. SISPA library preparation has the benefit of requiring less bench time to sequencing compared to the Twist CVRP protocol. Additionally, we found that the MinION's capability for long sequencing reads, was able to provide better coverage of the genome overall as compared to our attempt at SISPA on the MiSeq platform. Given the relative ease of library preparation and the technical difficulties of sequencing pathogens found within arthropod specimens without first isolating and culturing, SISPA sequencing on the MinION may be a less expensive alternative that is capable of field deployment. The downside is that there can be a higher rate of errors in reads when using the minION platform. While more data is required to determine the sensitivity of our genotyping RT-PCR assay, it seems likely that RT-PCR will be sufficient for providing not only screening of JEV but also typing of positive samples.

Future investigations might be able to implement the use of bioassays for JEV specific biomarkers as they could serve as rapid and cost-effective options to consider for screening because most JEV antigen detection assays are electrochemical-based, expensive, difficult to fabricate, required skilled handling, and are not portable point of care devices (29). Our laboratory is currently geared toward mass surveillance on a plethora of various arthropod vectored pathogens. In order to support the needs of our laboratory's mission, RT-PCR currently grants the laboratory a greater range of pathogen detection.

## Conclusion

This study demonstrates that utilization of the Direct-zol-96 MagBead RNA kit with the KingFisher Flex for automated nucleic acid isolation coupled with RT-qPCR techniques on the ABI 7500 remain the most sensitive, fastest, and cost-effective method for detection and genotyping of JEV in *Cx. tritaeniorhynchus* samples. These methods are especially useful when processing large batches of samples. For broader passive pathogen discovery, other sequencing based techniques such as SISPA or Twist panels may be appropriate depending on the goal of the laboratory, as these methods do not require target specific reagents and interpretation of results is only limited by the availability of viral genomic data. SISPA in particular has the potential to be performed in the field for passive detection of novel or emerging pathogens, but suffers from the lack of sequencing depth acquired through random amplification. The Twist panel shows greater promise for broad detection and potential genotyping despite the extended bench time required to prepare and sequence the hybridized libraries.

## Data availability statement

The datasets presented in this study can be found in online repositories. The names of the repository/repositories and accession number(s) can be found at: https://www.ncbi.nlm.nih.gov/, PRJNA894324.

## Author contributions

GC and KT conceived and designed the experiments, contributed reagents, materials, and analysis tools. GC, KW, EZ, AI, and ZH performed the experiments. GC, KW, and KT analyzed the data. GC, KW, EZ, AI, ZH, and KT wrote the paper. All authors read and approved the manuscript.

## Funding

This study was funded in part by the Armed Forces Health Surveillance Division (AFHSD), Global Emerging Infections Surveillance (GEIS) Branch, ProMIS ID P0009_21_OT and P0046_22_OT.

## Conflict of interest

The authors declare that the research was conducted in the absence of any commercial or financial relationships that could be construed as a potential conflict of interest.

## Publisher's note

All claims expressed in this article are solely those of the authors and do not necessarily represent those of their affiliated organizations, or those of the publisher, the editors and the reviewers. Any product that may be evaluated in this article, or claim that may be made by its manufacturer, is not guaranteed or endorsed by the publisher.

## Author disclaimer

The views expressed in this presentation are those of the authors and do not necessarily reflect the official policy of the Department of the Army, Department of Defense, or the U.S. Government.

## References

[1] Li M-H Fu S-H Chen W-X Wang H-Y Guo Y-H Liu Q-Y. Genotype V Japanese encephalitis virus is emerging. PLoS Negl Trop Dis. (2011) 5:e1231. 10.1371/journal.pntd.0001231 21750744 PMC3130007

[2] Pan XL Liu H Wang HY Fu SH Liu HZ Zhang HL. Emergence of genotype I of Japanese encephalitis virus as the dominant genotype in Asia. J Virol. (2011) 85:9847–53. 10.1128/JVI.00825-11 21697481 PMC3196406

[3] Oliveira ARS Cohnstaedt LW Noronha LE Mitzel D McVey DS Cernicchiaro N. Perspectives regarding the risk of introduction of the Japanese Encephalitis Virus (JEV) in the United States. Front Vet Sci. (2020) 7:48. 10.3389/fvets.2020.00048 32118069 PMC7019853

[4] Auerswald H Maquart PO Chevalier V Boyer S. Mosquito vector competence for Japanese Encephalitis Virus. Viruses. (2021) 13:1154. 10.3390/v13061154 34208737 PMC8234777

[5] Lindahl J Chirico J Boqvist S Thu HTV Magnusson U. Occurrence of Japanese Encephalitis Virus mosquito vectors in relation to urban pig holdings. Am Soc Trop Med Hyg. (2012) 87:1076–82. 10.4269/ajtmh.2012.12-0315 23033401 PMC3516078

[6] Ricklin ME Garcìa-Nicolàs O Brechbühl D Python S Zumkehr B Posthaus H. Japanese encephalitis virus tropism in experimentally infected pigs. Vet Res. (2016) 47:34. 10.1186/s13567-016-0319-z 26911997 PMC4765024

[7] Infectious Disease Surveillance Center and National Institute of Infectious Disease. Japanese Encephalitis, Japan, 2007-2016. Infectious Agents Surveillance Report. (2017). Available online at: https://www.niid.go.jp/niid/images/idsc/iasr/38/450e.pdf

[8] Choi MB Lee W-G Kang HJ Yang S-C Song BG Shin E-H. Seasonal prevalence and species composition of mosquitoes and chigger mites collected from Daegu, Gunwi and Sangju in South Korea, 2014. J Ecol Environ. (2017) 41:15. 10.1186/s41610-017-0030-7

[9] Faizah AN Kobayashi D Amoa-Bosompem M Higa Y Tsuda Y Itokawa K. Evaluating the competence of the primary vector, Culex tritaeniorhynchus, and the invasive mosquito species, Aedes japonicus japonicus, in transmitting three Japanese encephalitis virus genotypes. PLoS Negl Trop Dis. (2021) 14:e8986. 10.1371/journal.pntd.0008986 33370301 PMC7793266

[10] Amicizia D Zangrillo F Lai PL Iovine M Panatto D. Overview of Japanese encephalitis disease and its prevention. Focus on IC51 vaccine (IXIARO^®^). J Prevent Med Hyg. (2018) 59:E99–107. 10.15167/2421-4248/jpmh2018.59.1.962 29938245 PMC6009073

[11] Zhang B He Y Xu Y Mo F Mi T Shen QS. Differential antiviral immunity to Japanese encephalitis virus in developing cortical organoids. Cell Death Dis. (2018) 9:719. 10.1038/s41419-018-0763-y 29915260 PMC6006338

[12] World Health Organization. Japanese Encephalitis. (2019). Available online at: https://www.who.int/news-room/fact-sheets/detail/japanese-encephalitis

[13] Lobigs M Pavy M Hall RA Lobigs P Cooper P Komiya T. An inactivated Vero cell-grown Japanese encephalitis vaccine formulated with Advax, a novel inulin-based adjuvant, induces protective neutralizing antibody against homologous and heterologous flaviviruses. J Gen Virol. (2010) 91:1407–17. 10.1099/vir.0.019190-0 20130134 PMC2888167

[14] Erra EO Askling HH Yoksan S Rombo L Riutta J Vene S. Cross-protective capacity of Japanese encephalitis (JE) vaccines against circulating heterologous JE virus genotypes. Clin Infect Dis. (2013) 56:267–70. 10.1093/cid/cis883 23074319 PMC3526254

[15] Sanborn MA Wuertz KM Kim H-C Yang Y Li T Pollett SD. Metagenomic analysis reveals Culex mosquito virome diversity and Japanese encephalitis genotype V in the Republic of Korea. Mol Ecol. (2021) 30:5470–87. 10.1111/mec.16133 34418188

[16] Mulvey P Duong V Boyer S Burgess G Williams DT Dussart P. The ecology and evolution of Japanese encephalitis virus. Pathogens. (2021) 10:1534. 10.3390/pathogens10121534 34959489 PMC8704921

[17] Eick-Cost AA Hu Z Klein TA Putnak RJ Jarman RG. Seroconversion to Japanese Encephalitis virus among U.S. infantry forces in Korea. Am Soc Trop Med Hyg. (2015) 93:1052–4. 10.4269/ajtmh.15-0307 26240157 PMC4703268

[18] Centers for Disease Control and Prevention. Japanese Encephalitis Vaccine | Japanese Encephalitis. (2019). Available online at: https://www.cdc.gov/japaneseencephalitis/vaccine/index.html

[19] Calvert AE Dixon KL Delorey MJ Blair CD Roehrig JT. Development of a small animal peripheral challenge model of Japanese encephalitis virus using interferon deficient AG129 mice and the SA14-14-2 vaccine virus strain. Vaccine. (2014) 32:258–64. 10.1016/j.vaccine.2013.11.016 24252694 PMC3910511

[20] Shao N Li F Nie K Fu SH Zhang WJ He Y. TaqMan real-time RT-PCR assay for detecting and differentiating Japanese encephalitis virus. Biomed Environ Sci. (2018) 31:208–14. 10.3967/bes2018.026 29673443

[21] Wright MS Stockwell TB Beck E Busam DA Bajaksouzian S Jacobs MR. Sispa-Seq for rapid whole genome surveys of bacterial isolates. Infect Genet Evol. (2015) 32:191–8. 10.1016/j.meegid.2015.03.018 25796360 PMC5556377

[22] Chrzastek K Lee D Smith D Sharma P Suarez DL Pantin-Jackwood M. Use of Sequence-Independent, Single-Primer-Amplification (SISPA) for rapid detection, identification, and characterization of avian RNA viruses. Virology. (2017) 509:159–66. 10.1016/j.virol.2017.06.019 28646651 PMC7111618

[23] Heng L. Seqtk-1.3 (r106): Toolkit for Processing Sequences in FASTA/Q Formats. (2018). Available online at: https://github.com/lh3/seqt

[24] Magoč T Salzberg SL. FLASH: fast length adjustment of short reads to improve genome assemblies. Bioinformatics. (2011) 27:2957–63. 10.1093/bioinformatics/btr507 21903629 PMC3198573

[25] Shabardina V Kischka T Manske F Grundmann N Frith MC Suzuki Y. NanoPipe—a web server for nanopore MinION sequencing data analysis. GigaScience. (2019) 8:giy169. 10.1093/gigascience/giy169 30689855 PMC6377397

[26] Milne I Stephen G Bayer M Cock PJA Pritchard L Cardle L. Using tablet for visual exploration of second-generation sequencing data. Brief Bioinform. (2013) 14:193–202. 10.1093/bib/bbs012 22445902

[27] Verdonschot PFM Besse-Lototskaya AA. Flight distance of mosquitoes (Culicidae): a metadata analysis to support the management of barrier zones around rewetted and newly constructed wetlands. Limnologica. (2014) 45:69–79. 10.1016/j.limno.2013.11.002

[28] Cao L Fu S Gao X Li M Cui S Li X. Low protective efficacy of the current Japanese encephalitis vaccine against the emerging genotype 5 Japanese encephalitis virus. PLoS Negl Trop Dis. (2016) 10:e0004686. 10.1371/journal.pntd.0004686 27139722 PMC4854478

[29] Roberts A Gandhi S. A brief review on novel biomarkers identified and advanced biosensing technologies developed for rapid diagnosis of Japanese Encephalitis Virus. Proc Indian Natl Sci Acad. (2022). 10.1007/s43538-022-00113-1. [Epub ahead of print].

