# Supplementary material for: Method comparison for Japanese encephalitis virus detection in samples collected from the Indo-Pacific region
Source: Front Public Health. 2022 Nov 24;10:1051754. doi: 10.3389/fpubh.2022.1051754 (PMC9730272; doi:10.3389/fpubh.2022.1051754)
Supplement: Supplementary file 1 [file Table_1.docx]

| **MagPix Net MFI** | | | | |
| --- | --- | --- | --- | --- |
| **Sample** | **JEV** | **IC** | **FC** | **NSBC** |
| A21.2333 | 68 | 5576 | 3259 | 68 |
| A21.2437 | 79 | 5543 | 3065 | 75 |
| A21.2560 | 75 | 5606 | 3292 | 76 |
| A21.2711 | 75 | 5527 | 3615 | 75 |
| A21.2838 | 77 | 5575 | 3472 | 79 |
| A21.2886 | 72 | 5550 | 3239 | 74 |
| A21.3160 | 80 | 5560 | 3917 | 79 |
| A21.3171 | 78 | 5522 | 4333 | 74 |
| A21.3232 | 84 | 5570 | 5331 | 78 |
| A21.3389 | 82 | 5531 | 5109 | 86 |
| A21.3401 | 75 | 5555 | 3820 | 77 |
| A21.3402 | 75 | 5490 | 3612 | 76 |
| A21.3404 | 73 | 5560 | 3474 | 76 |
| A21.3465 | 69 | 5580 | 3362 | 75 |
| A21.3577 | 71 | 5515 | 3010 | 77 |
| A21.3585 | 72 | 5597 | 3121 | 73 |
| A21.3587 | 72 | 5516 | 4204 | 74 |
| A21.3675 | 65 | 5556 | 3213 | 71 |
| A21.3678 | 71 | 5526 | 3027 | 74 |
| A21.3682 | 64 | 5484 | 2760 | 74 |
| A21.3683 | 68 | 5500 | 2651 | 71 |
| GU-22-58-20 | 62 | 5498 | 3631 | 62 |
| GU-22-58-21 | 59 | 5567 | 2746 | 60 |
| MBP_CD | 4041 | 5607 | 3059 | 91 |
| Extraction Control | 81 | 5567 | 4127 | 85 |
| JEV G3 1:1,000 | 564 | 5654 | 2892 | 80 |
| MBP_CD | 8332 | 5655 | 8795 | 98 |
| Extraction Control | 97 | 5542 | 9619 | 84 |

**Supplementary Table 1 -** MagPix Mega Mosquito-borne Pathogen MultiFLEX® Panel analytes and their respective net MFI values. (JEV - Japanese Encephalitis; IC - internal control; FC - fluorescence control; NSBC - non-specific binding control)
