# Supplementary figures and images for: Method comparison for Japanese encephalitis virus detection in samples collected from the Indo-Pacific region
Source: Front Public Health. 2022 Nov 24;10:1051754. doi: 10.3389/fpubh.2022.1051754 (PMC9730272; doi:10.3389/fpubh.2022.1051754)

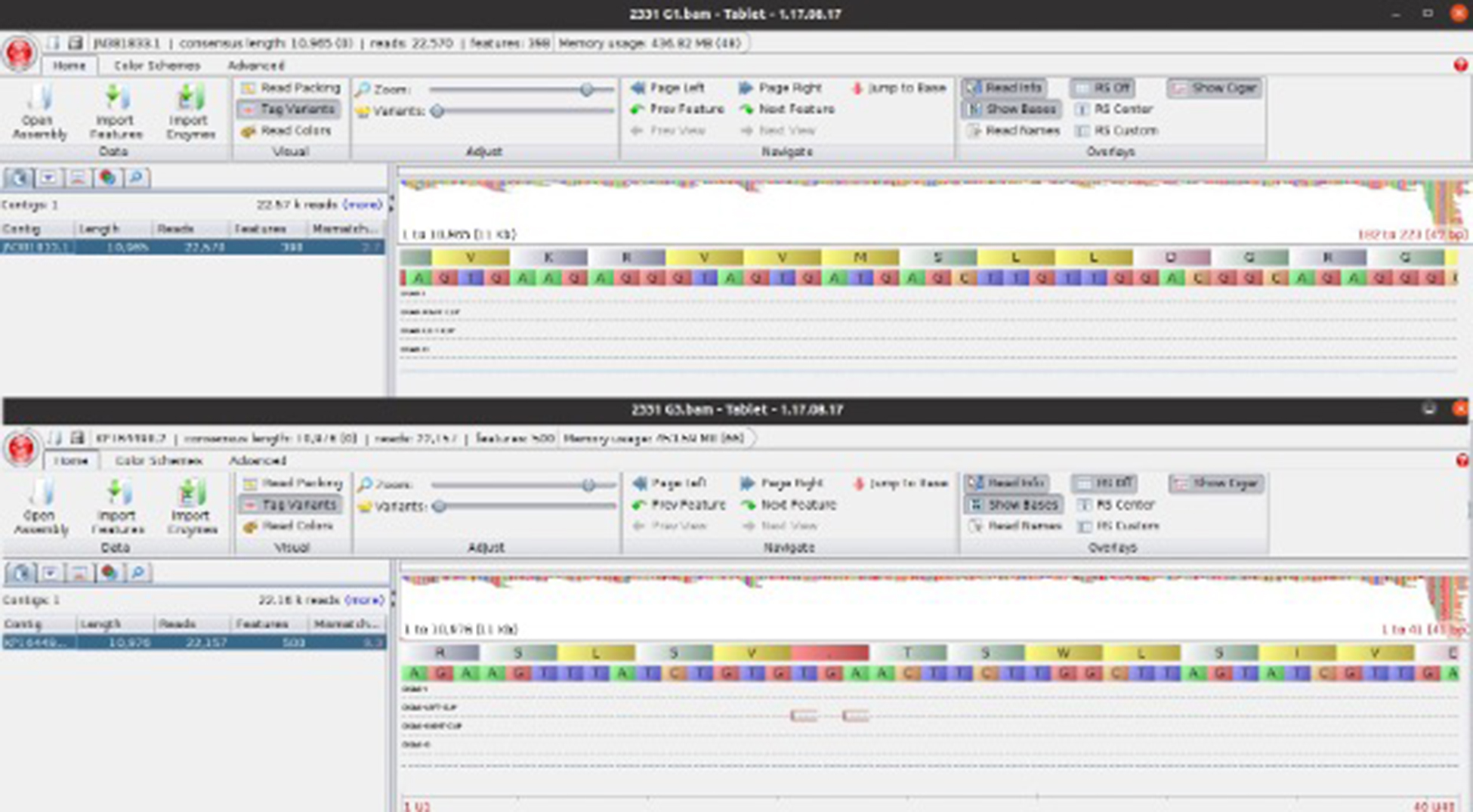

Supplement: Supplementary file 2 [file Image_1.jpg]
